# Nest predation risk influences a cavity-nesting passerine during the post-hatching care period

**DOI:** 10.1038/srep31989

**Published:** 2016-08-24

**Authors:** Jongmin Yoon, Byung-Su Kim, Eun-Jin Joo, Shi-Ryong Park

**Affiliations:** 1 Ecological Institute for Oriental Stork, Korea National University of Education, Cheongju 28173, Republic of Korea; 2 Department of Biology Education, Korea National University of Education, Cheongju 28178, Republic of Korea

## Abstract

Some nest predators visually assess parental activities to locate a prey nest, whereas parents modify fitness-related traits to reduce the probability of nest predation, and/or nestlings fledge early to escape the risky nest environment. Here, we experimentally tested if the parental and fledging behaviours of oriental tits (*Parus minor*) that bred in the nest-box varied with cavity conditions associated with nest predation risk during the nestling period. The entrance of experimental nest-boxes was enlarged to create a long-term risk soon after clutch competition. A short-term risk, using simulated playbacks with a coexisting control bird and avian nest predator sound, was simultaneously applied to the nest-boxes whether or not the long-term risk existed. We found that the parents reduced their hourly feeding trips, and the nestlings fledged early with the long-term risk, although the nest mortality of the two nest-box types was low and did not differ. While this study presents a portion of prey–predator interactions with the associated uncertainties, our results highlight that the entrance size of cavities for small hole-nesting birds may play an important role in determining their fitness-related traits depending upon the degree of perceived risk of nest predation.

Nest predation is one of key drivers of selection for small breeding passerines, in which the parents, offspring, and nest predators may interact to escape the risky nest environment that can impair the reproductive success of prey birds^1^. Parental care is presumed to have evolved from trade-offs between the fitness-related costs and benefits of providing care to facilitate offspring survival^2^,^3^,^4^. Parents may take advantage of phenotypic plasticity^5^ depending upon variations in environmental conditions to determine the optimal levels of these trade-offs^6^. In altricial birds, provisioning nestlings with food should impose a cost when parental activity draws the attention of predators to the nest location^7^,^8^,^9^; parents may compensate by reducing brood size to lower nest visitation, increase per-trip food load, and decrease allofeeding (e.g. male incubation feeding), whereas nestlings may fledge early to escape the risky nest environment^10^.

Cavity nesters experience relatively lower risks of nest predation due to limited predator access, compared to open-cup nesters^11^,^12^,^13^,^14^. The breeding opportunities of weak or non-excavators may be critically limited by unpredictable and inappropriate nest sites, resulting in large clutch sizes^15^,^16^,^17^,^18^. Weak or non-excavators must cope with variations in cavity conditions, such as entrance size and cavity depth, as well as the attentions of diverse nest predators, compared to strong excavators. Therefore, cavity-nesting birds that lack the propensity to excavate can be more vulnerable to nest predation depending on cavity quality than excavators^8^,^19^,^20^, and with respect to the contexts of nest predators^1^.

Parental activity at the nest can attract nest predators, resulting in reduced parental nest activity during the incubation and nestling periods^7^. To conceal the nest via reducing parental nest visits, selection may favour high nest attentiveness, a low feeding rate with large food load, and/or high vigilance while entering and exiting the nest^9^. Alternatively, faster nestling growth with decreased per-nestling food provisioning was associated with higher nest predation risk presumably due to differential allocation of resources while growing^21^. Otherwise, the rate of nestling growth can be reduced due to decreased feeding effort of parents when the predation risk refers to their own risk rather than that of their brood^22^. Here, energy provided to nestlings in the form of food must be allocated to the competing demands of development, thermoregulation, and begging behaviour^23^,^24^,^25^,^26^.

The main goal of the present study was to examine the response of cavity-nesting oriental tits (*Parus minor*) to manipulated risks of nest predation during the post-hatching period. Here, we simultaneously applied two types of nest predation risk: “short-term” (simulation of predator presence) versus “long-term” (manipulation of cavity entrance condition; Fig. 1). The short-term risk may become less effective in the presence of high predator activity, whereas the long-term risk may be less crucial when the degree of predator activity is low^27^,^28^. Furthermore, the effect of short-term risk has been tested more frequent than that of long-term risk^29^,^30^,^31^.

## Results

Differences in parental feeding behaviour and nestling period of oriental tits with similar brood sizes were found in the presence of long-term risk (i.e. nest-box with control versus enlarged entrance), but no effect of short-term risk was observed (Fig. 2). The variation in female nest attentiveness (%) was not explained by any risk factors (mixed model: nest *F*_1,20.8_ = 1.42, *P* = 0.25; playback *F*_1,22.4_ = 0.20, *P* = 0.66; nest × playback *F*_1,21.9_ = 1.04, *P* = 0.32; temperature *F*_1,41.9_ = 0.06, *P* = 0.80; brood *F*_1,21.5_ = 0.27, *P* = 0.61). The long-term risk affected the number of parental feeding trips per hour (mixed model, nest *F*_1,20.9_ = 5.25, *P* = 0.03; playback *F*_1,22.2_ = 1.13, *P* = 0.30; nest × playback *F*_1,21.7_ = 1.40, *P* = 0.25; temperature *F*_1,38.2_ = 6.37, *P* = 0.02; brood *F*_1,21.3_ = 6.71, *P* = 0.02; Fig. 2a). Specifically, the parents in the nest-box with an enlarged entrance (19.16 ± 2.10 trips/hr) made 26% fewer feeding trips per hour than those in the nest-box with a control entrance (25.96 ± 2.10 trips/hr), and the overall feeding rate decreased with increasing ambient temperature (slope estimate: −0.73 ± 0.28) and increased with increasing brood size (slope estimate: 4.28 ± 1.65). The parents exhibited more-vigilant behaviour in the nest-box with an enlarged entrance (2.07 ± 0.41%) than in that with a control entrance (0.90 ± 0.41%), but this was marginally significant (mixed model, nest *F*_1,20.1_ = 4.12, *P* = 0.06; playback *F*_1,21.3_ = 0.28, *P* = 0.60; nest × playback *F*_1,20.9_ = 2.93, *P* = 0.10; temperature *F*_1,36.0_ = 2.05, *P* = 0.16; brood *F*_1,20.5_ = 0.88, *P* = 0.36; Fig. 2b). We did not observe any complete depredation during the incubation and nestling periods, although one of 12 nest-boxes of each type experienced only partial nest depredation (i.e. missing one or two nestlings with disturbed nesting materials). The nestlings in the nest-box with an enlarged entrance (15.67 ± 0.74 days) fledged 1.83 ± 0.83 days earlier than those in the nest-box with a control entrance (17.50 ± 0.38 days) (*t* test, *t*_22_ = 2.20, *P* = 0.04; Fig. 3).

## Discussion

We examined the influence of cavity condition of oriental tits’ nest-boxes on parental and fledging behaviour (Fig. 2), and nest success by manipulating the short- and long-term risks; Fig. 3) risks of nest predation. Our results support in part the hypothesis that the parents modified fitness-related traits to reduce the probability of nest detection (Fig. 2), and the nestling period was shortened in the risky nest-box with an enlarged entrance (Fig. 3)^7^. However, the short-term risk was less effective in the context of high predator activity compared to the long-term risk. Magpies and jays as major nest predators that can easily depredate the nest-box with an enlarged entrance but not with the control may have been locally abundant, so the magpie sound stimuli might be insufficient to provoke detectable responses of parents; this finding during the nestling period was similar to that during the incubation period in our study site with high nest predator activity.

The long-term risk of nest predation increased with reduced parental feeding rates in oriental tits; therefore, this long-term risk did not influence fledging success directly when nest predation was minimal in both types of nest-boxes. In general, the degree of nest predation increases with the progress of breeding stages because the number of nest trips made by parents is likely to increase from the incubation period to the nestling period at the expense of nestling growth and survival, after controlling for nest-site effects^32^. For example, our previous study showed significant differences in the parental behaviour and nest predation rate of two cavity-nesting species (i.e. marsh tits, *Poecile palustris*, versus oriental tits) breeding in nest-boxes with control versus enlarged entrances during the incubation period. Marsh tits with a high frequency of nest trips (both male’s for feeding the incubating females and female’s for self-feeding) and lacked plasticity in their incubation behaviour independent of perceived predation risks experienced relatively high nest mortality during the incubation period. Moreover, few nests of marsh tits survived to fledging in the nest-box with an enlarged entrance; oriental tits, however, tended to experience a low nest predation rate throughout the breeding season. Thus, their anti-predator plasticity (i.e. a steeper slope of reaction norms)^9^ in nest visitation and vigilance at the nest-box might play a role in lowering nest predation throughout the breeding season, independent of nest-box type^31^.

Discrepancies between nestling development and fledging time may depend on the relative degrees of nest versus fledgling predation risks even along the altricial-precocial spectrum in birds^33^,^34^. Length of the nestling period had a strong influence on fledgling mortality^35^,^36^. The nestling period of oriental tits was shorter in the nest-box with an enlarged entrance, compared to that in the absence of this long-term risk, although the parental feeding rate was reduced in the former condition. This result supports the hypothesis that the nestling period is shortened in response to increased nest predation risk^10^,^13^. It is uncertain whether oriental tit nestlings fledge prematurely to escape the relatively risky nest environment. In general, altricial nestlings should allocate resources from parental feeding and brooding mostly into development and endothermy^26^,^37^, depending upon the internal and external environments (e.g. nest versus fledgling predation risks). It is possible that nest predation risk is higher than fledgling predation risk in the context of open-cup nesters; in contrast, the former risk should be much lower than the latter risk in cavity nesters^34^,^35^. Alternatively, the existence of a carry-over effect of incubation temperature and/or rhythm made by females on nestling growth under high nest predation should be determined^34^. This negative effect on nestlings at fledging is likely to include premature development^34^ with increased metabolic rate^38^ or changes in early avian phenotypic development through stress-induced increases in plasma corticosterone levels^39^,^40^. Therefore, this carry-over effect during the incubation period should be disentangled from the effect of post-hatching care, such as parental feeding and brooding, during the nestling period^41^.

In the present study, the behavioural plasticity in parental activities at the nest improved the rate of nest survival in non-excavating cavity nesting oriental tits, but reduced the duration of the nestling period in the presence of a long-term risk of nest predation. Prior to nesting, nest-site selection may be more adaptive than behavioural plasticity for non-excavating nesters as the cavity conditions for nesting vary in nature. Available nesting cavities are more abundant than previously thought, and cavity nesters may not face a shortage of holes^42^,^43^. However, secure cavities may be counterbalanced by limited supply of natural ones and interspecific competition for acquisition^12^. Otherwise, anti-predatory parental care under high risk conditions may either compensate for nest predation costs resulting in reduced offspring mortality^44^ or indirectly increase mortality through reduced care^45^. While the results represent a reaction norm of one species under variation in nest predation risks with associated uncertainty (e.g. a comparison of nest versus fledging predation risks in cavity-nesters), we suggest the need for additional hypotheses for the ecological correlates and evolutionary drivers of intra- and interspecific variation in incubating and post-hatching care behaviour with the propensity for excavation in cavity-nesting birds. Our behavioral observations were nevertheless limited to a short period by keeping the two nestling ages. Additional studies across the nestling ages with increased sampling are required to come to a robust conclusion to generalize our findings.

## Methods

### Study site and species

**Figure 1:**
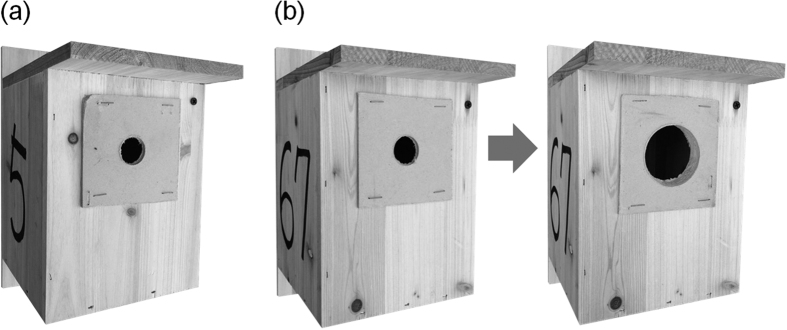
Manipulation of the nest-box entrance as a long-term nest predation risk for oriental tits: (**a**) control nest box with a 2.6 cm diameter entrance and (**b**) treatment nest box with a 5.5 cm diameter entrance (after clutch completion) changed from the 2.6 cm (before clutch completion) diameter entrance by switching the front panel for one a larger entrance hole.

**Figure 2:**
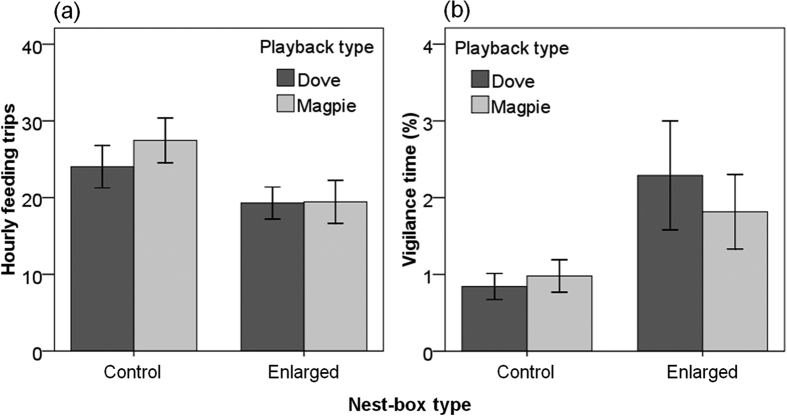
Behavioural plasticity in (**a**) the number of parental feeding trips per hour and (**b**) vigilance time (%) of nest-box breeding oriental tits (*Parus minor*) in response to the long-term (nest-box with control or enlarged entrance) and short-term (sound playback using non-predatory dove or nest-predatory magpie) risk of nest predation. Bars denote means of nest-boxes with control (12 nests) versus enlarged (12 nests) entrance ± 1·SE.

**Figure 3:**
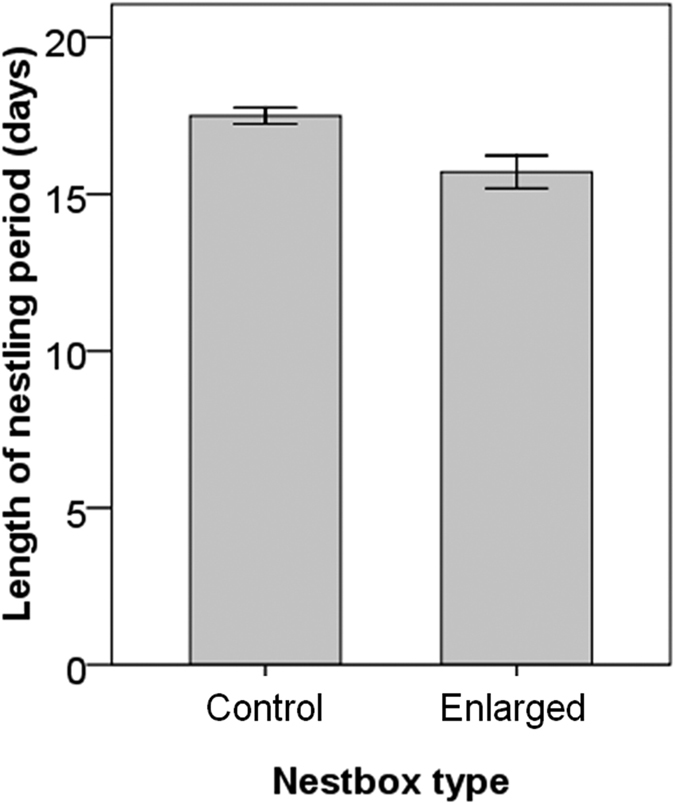
Differences in the nestling period length (days) of oriental tits in response to the long-term risk of nest predation. Bars denote means of nest-boxes with control (11 nests) versus enlarged (11 nests) entrance ± 1·SE.

We studied the interactions between oriental tits (24 nests) and corvids as avian nest predators inhabiting the campus of the Korea National University of Education, Cheongju, Republic of Korea (N 36° 36′ 33.58″, E 127° 21′ 34.40″) during March-June of 2014. The breeding habitat (85 ha) was composed of patchy forest with mixed deciduous and coniferous trees interspersed with buildings. Previously, we monitored about 25–30 pairs of oriental tits each year in our study site. We installed 50 nest boxes (2 m off the ground) during December 2010. Nest boxes (11 cm long × 16 cm deep × 21 cm high), with an initial 2.6 cm diameter entrance hole, were apart from each other by at least 100 m (Fig. 1). The preliminary study showed oriental tits, marsh tits, and coal tits (*Periparus ater*) shared this entrance size in our study site (J. Yoon, personal observation). We captured adults using mist nets and banded them with a unique combination of three coloured leg bands and a numbered aluminium band (National Institute for Biological Resources) to identify individuals. Sex was determined based on the presence of the cloacal protuberance for males and the brood patch for incubating females^46^. Our preliminary results indicated that female oriental tits laid 7–14 eggs per clutch, incubated eggs, and brooded the nestlings, and both parents fed them for 15–18 days prior to fledging. They were also observed as non-excavators nesting in trees or artificial cavities in our study site^46^. All nest-boxes were checked every 2 days to confirm breeding stage and nest depredation.

Corvids as generalist omnivores are efficient nest predators that affect many bird species and use parental nest activities as a visual cue to locate prey nests^47^,^48^,^49^,^50^,^51^. In our study site, we have monitored five potential nest predators for breeding birds, including four avian predators, such as Eurasian magpies (*Pica pica*), Eurasian jays (*Garrulus glandarius*), Azure-winged magpies (*Cyanopica cyanus*), and gray-headed woodpeckers (*Picus canus*), as well as the red squirrel (*Sciurus vulgaris*) as a mammalian predator. However, grey-headed woodpeckers and red squirrels were rarely detected at the study site, where Eurasian magpies (88%) were detected most frequently, followed by Eurasian jays (8%) and Azure-winged magpies (4%). Our preliminary results indicated that the activity of these avian nest predators also appeared to respond to cavity condition and the parental activity level of cavity-nesting parids during the incubation period.

### Long-term nest predation risk

We manipulated the entrance size of the nest-boxes soon after the clutch was completed (Fig. 1)^20^,^31^. The original entrance hole to all nest boxes was 5.5 cm in diameter, and we covered the entrance hole with a 7 × 7 cm panel with a 2.6 cm diameter hole prior to breeding (December-January). Once the parids completed their clutches, we detached the panel from the treatment nest-boxes with a 5.5-cm entrance hole (Fig. 1b; 12 nests), but not from the control nest-boxes with a 2.6 cm entrance hole during the incubation and nestling periods (Fig. 1a; 12 nests). The treatment nest-box condition with the 5.5 cm entrance hole was based on the potential use of cavities excavated by one of the most common primary excavators, such as grey-headed woodpeckers, at our study site (i.e. imitation of woodpecker’s cavity). We also examined the effect of differential entrance size on nest-box internal temperature from April to May of 2013, using a temperature data logger (iButton DS1921G, Maxim Integrated, San Jose, CA, USA) placed in one of each unoccupied nest-box type. The internal condition (i.e. per-hour temperature for approximately 2 months) did not differ significantly between the control and enlarged nest boxes after manipulating entrance size. Additionally, brood size (control: 9.33 ± 0.33; enlarged: 9.42 ± 0.19; effect size = 0.09) did not differ between the two nest-box types where the overall rates of hatching success with 96% did not differ during the study period (effect size = 0.13).

### Short-term nest predation risk

Oriental tits that nested in the control or enlarged nest-boxes with different entrance-hole sizes were simultaneously exposed to two sessions of simulated sound playbacks to examine the effect of short-term risk of nest predation on parental behaviour. We performed sound playback sessions using the sounds of a non-nest predator (oriental turtle-dove, *Streptopelia orientalis*) and the most common nest predator (Eurasian magpie) near the nest-box. Oriental turtle-doves were abundant and less likely to compete with the parid species for foraging and nesting at the study site^52^. The predator-simulating loudspeaker was placed approximately 5 m from the nest-box. We used an identical portable speaker (frequency response: 80 Hz-16 kHz; K2000, Laconia Inc., Seoul, Republic of Korea) connected to an MP3 player (T8, Iriver Ltd., Seoul, Republic of Korea) with the same volume level to standardise playback stimuli for all nest boxes. The camcorder was set to start at least 5 min after the placement and initiation of the loudspeaker to minimise modification of parental behaviour in the nest video data. We conducted two playback sessions per nest-box on day 9 and 10 during the nestling period (i.e. hatching day was day 0): one session using oriental turtle-dove sounds and the other using Eurasian magpie sounds. The two playback sessions per nest-box were at least 24 h apart, and the order of non-nest predator and nest predator sessions was randomised. The use of randomly selected sounds (a random order and repetition of one of three different 10 s sounds for magpies and doves and one 30 s silence produced by an MP3 player using its shuffle and repeat functions) was designed for non-nest predator and nest predator stimuli^53^.

### Parental behaviour

All focal birds that bred in the nest-boxes with and without the long-term risk were exposed to the short-term risk to assess parental behaviours and the plasticity thereof (see above for timing of videotaping). We videotaped each nest box using a digital camcorder (HDR-CX12, Sony Corp., Tokyo, Japan) without human disturbance (5 m from the nest box) to collect behavioural data during the nestling period in the presence of a mixture of short-term and/or long-term risks of nest predation. Video recording was conducted for 3 h on day 9 and 10 during the nestling period (09:00–12:00 KST) during exposure to the long-term risk (see above). We transcribed the recorded files using media player software (GOM Media Player, Gretech Corp., Republic of Korea). We analysed parental behaviours including the proportion of female’s time spent at the nest (%; female’s nest attentiveness) and the number of nest trips by the parents during the total observation time. We also scored the proportion of parental vigilance time (%) from each video. This was calculated as the time spent scanning the surrounding area while perched either outside or inside the nest entrance hole)^31^.

### Statistical analyses

We analyzed female nest attentiveness (%), hourly feeding trips, and vigilance time (%) of the parents in relation to playback stimulus (dove versus magpie sounds; short-term risk), nest-box type (control versus enlarged entrance; long-term risk), and the two-way interaction as fixed effects (playback × nest), using a linear mixed model with restricted maximum likelihood (REML). We also included ambient temperature (°C) and brood size (the number of nestlings per nest) as covariates in the model, which were likely to influence parental behaviours such as feeding and brooding nestlings^54^. Nest ID was included as a random effect to control for repeated measurements of the same nest boxes for the short-term risk. Fisher’s least significant difference (LSD) was used to examine significant interaction effects. Otherwise, a two-sample *t* test was used to examine the effect of the long-term risk on the length of nestling period. All statistical analyses were performed using SPSS ver. 16.0 (SPSS Inc., Chicago, IL, USA). Data are presented as means ± 1·SE.

### Ethical statement

 All experimental protocols were approved by the Animal Care and Use Committees at the Korea National University of Education and performed in accordance with the relevant guidelines and regulations of the Ecological Institute for Oriental Stork in the Korea National University of Education.

## Additional Information

**How to cite this article**: Yoon, J. *et al*. Nest predation risk influences a cavity-nesting passerine during the post-hatching care period. *Sci. Rep.* **6**, 31989; doi: 10.1038/srep31989 (2016).
